# I-Motif/miniduplex hybrid structures bind benzothiazole dyes with unprecedented efficiencies: a generic light-up system for label-free DNA nanoassemblies and bioimaging

**DOI:** 10.1093/nar/gkaa020

**Published:** 2020-01-17

**Authors:** Lili Shi, Pai Peng, Jiao Zheng, Qiwei Wang, Zhijin Tian, Huihui Wang, Tao Li

**Affiliations:** Department of Chemistry, University of Science and Technology of China, 96 Jinzhai Road, Hefei, Anhui 230026, China

## Abstract

I-motif DNAs have been widely employed as robust modulating components to construct reconfigurable DNA nanodevices that function well in acidic cellular environments. However, they generally display poor interactivity with fluorescent ligands under these complex conditions, illustrating a major difficulty in utilizing i-motifs as the light-up system for label-free DNA nanoassemblies and bioimaging. Towards addressing this challenge, here we devise new types of i-motif/miniduplex hybrid structures that display an unprecedentedly high interactivity with commonly-used benzothiazole dyes (e.g. thioflavin T). A well-chosen tetranucleotide, whose optimal sequence depends on the used ligand, is appended to the 5′-terminals of diverse i-motifs and forms a minimal parallel duplex thereby creating a preferential site for binding ligands, verified by molecular dynamics simulation. In this way, the fluorescence of ligands can be dramatically enhanced by the i-motif/miniduplex hybrids under complex physiological conditions. This provides a generic light-up system with a high signal-to-background ratio for programmable DNA nanoassemblies, illustrated through utilizing it for a pH-driven framework nucleic acid nanodevice manipulated in acidic cellular membrane microenvironments. It enables label-free fluorescence bioimaging in response to extracellular pH change.

## INTRODUCTION

As two important four-stranded DNA structures, G-quadruplexes (G4s) and i-motifs have proven able to form in telomeric DNAs as well as genic regions of human cells (1,2). They share some common features such as their reversible response to external stimuli (3–5) and interactivity with a number of ligands (6,7), allowing them to serve as powerful modulating systems for programmable DNA nanoassemblies (8,9). Meanwhile, G4s can be also employed together with ligands as the light-up systems for label-free bioanalysis (10,11) and cell imaging (12–15). However, i-motifs rarely do so due to their poor interactivity with the reported ligands (16,17) in the given physiological conditions (Supplementary Figure S1A). Since i-motif-modulated DNA nanoassemblies are favored in acidic lysosomal compartments (18,19) and cellular membrane microenvironments (20), it's of particular interest to offer i-motifs high interactivity with ligands even under complex cellular conditions. Toward this goal, here we seek to engineer novel highly effective i-motif hybrid structures for binding ligands via adding an overhang onto diverse i-motifs.

**Figure 1.**
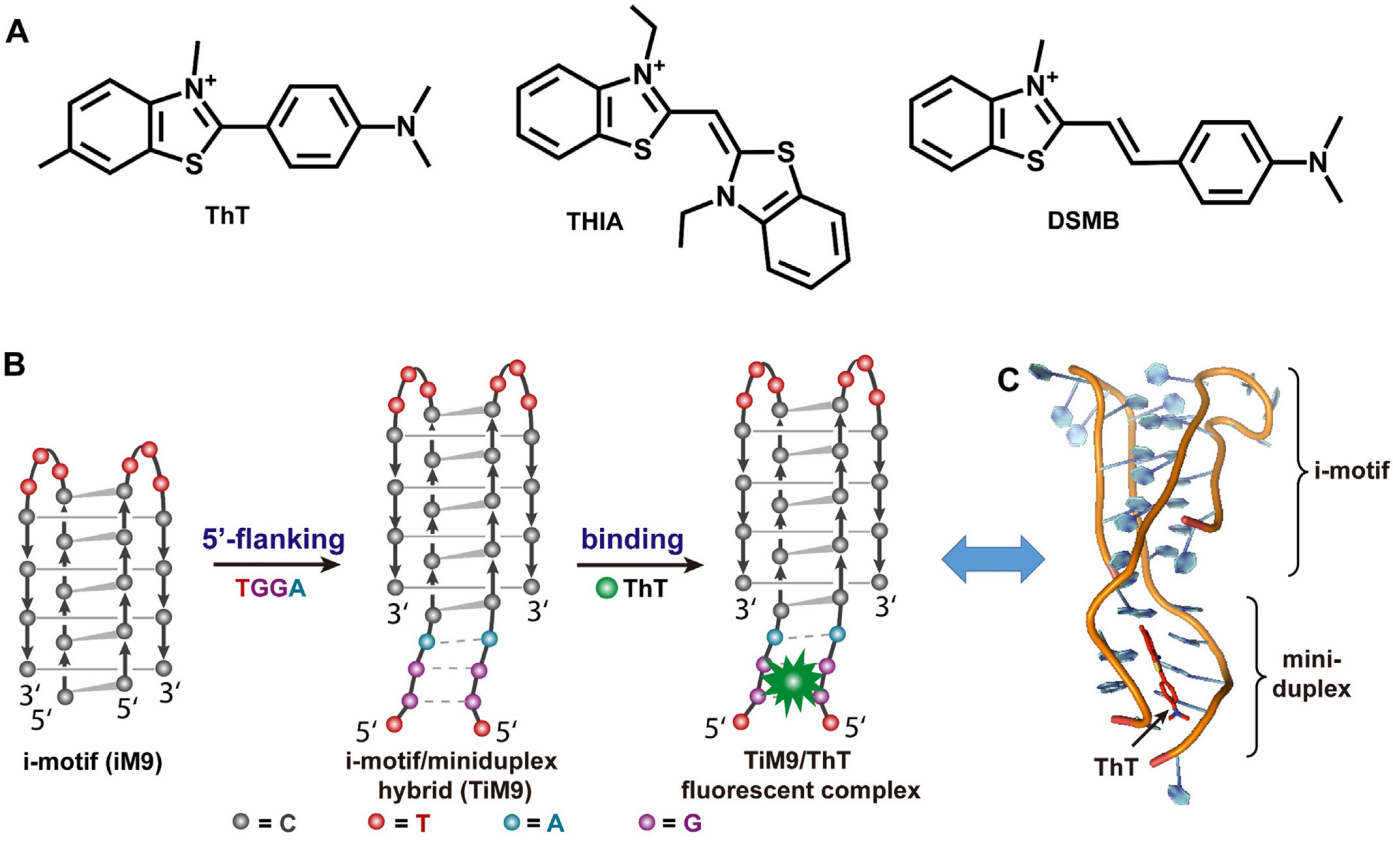
Schematic for engineering the ligand-interactive i-motif/miniduplex hybrid structures. (**A**) Molecular structures of three benzothiazole dyes used here. In the free state, these molecular rotors display weak fluorescence due to intramolecular charge transfer (21). (**B**) Structural alteration of a bimolecular i-motif C_5_T_3_C_4_ (iM9) reported previously (30). Extending the core structure of iM9 via 5′-flanking TGGA to obtain a hybrid structure (TGGAC_5_T_3_C_4_, TiM9) that binds ThT and enhances its fluorescence. (**C**) A MDS model of the TiM9/ThT complex.

Among DNA ligands, molecular rotors generally show a high signal-to-background ratio for lighting up diverse DNA structures due to their weak fluorescence at the twisted intramolecular charge-transfer state (21). One commonly-used type of molecular rotor is the benzothiazole dye such as thioflavin T (ThT) and derivatives (Figure 1A), which is usually employed as the G-quadruplex-specific fluorescent probe (12,14,22,23). In addition, ThT is proven insensitive to pH change (Supplementary Figure S1B), making it possible to utilize ThT as the fluorescent ligand in acidic cellular environments. Recently, ThT was also demonstrated able to bind homobase pairs, especially the G•G pairs, in parallel duplex DNAs (pdDNAs), accompanied by a sharp fluorescence enhancement (24,25). Since the i-motif core consists of two intercalated pdDNAs (26), we envisage that these pdDNAs may be extended with a few ThT-interactive homobase pairs. In fact, several noncanonical base pairs stably existing in parallel duplexes have been reported to extend the core structures of i-motifs (27,28). Hence, an i-motif hybrid structure with an appropriate overhang can in principle create a preferential site for binding ThT, providing a basis for building a new light-up system for i-motif-programmed DNA nanoassemblies (9,18,29,30).

With this idea in mind, herein we append a well-chosen tetranucleotide (TGGA) to the 5′ terminal of a bimolecular i-motif C_5_T_3_C_4_ (iM9) (30), as depicted in Figure 1B. We employ molecular dynamics simulations (MDS) to show that this 5′-overhang can form a very short parallel-stranded motif on the dimeric structure of iM9 (Figure 1C). Surprisingly, the resulting hybrid structure displays an unprecedentedly high capacity of binding ThT among all DNA forms reported so far (22,24,25,31–33). Varying the tetranucleotide sequence, similar phenomena are also observed when testing other molecular rotors (Figure 1A). This finding allows the hybrid to serve as a novel light-up system as well as a controlling unit for programmable DNA nanoassemblies, illustrated by utilizing it to functionalize a pH-driven framework nucleic acid (FNA) (34–36) nanodevice and in situ manipulate it on cellular membrane surface. It circumvents the need for fluorescent DNA labelling commonly used in other counterparts (20,37,38).

## MATERIALS AND METHODS

### Molecular rotors

Thioflavin T (ThT) and 3,3′-diethylthiacyanine (THIA) were purchased from Sigma-Aldrich (USA) and used without further purification. The concentration of aqueous ThT solution was quantified with UV-Vis spectroscopy as described previously (22). A recently reported compound 2-[4-(dimethylamino)styryl]-3-methyl-benzothiazole (DSMB) (39) was synthesized by methylating trans-2-[4-(dimethylamino)-styryl]benzothiazole (Sigma-Aldrich, USA) with iodomethane in DMF at 80°C for 24 h in a sealed flask, followed by HPLC purification and NMR, MS characterization (Supplementary Figure S2). ^1^H-NMR (500 MHz, DMSO) δ 8.30 (d, *J* = 10 Hz, 1H), 8.08 (d, *J* = 5 Hz, 1H), 8.02 (d, *J* = 15 Hz, 1H), 7.92 (d, *J* = 5 Hz, 2H), 7.77 (t, *J* = 15 Hz, 1H), 7.67 (t, *J* = 20 Hz, 1H), 7.6 (d, *J* = 15 Hz, 1H), 6.82 (d, *J* = 5 Hz, 2H), 4.22 (s, 3H), 3.10 (s, 6H). ^13^C-NMR (101 MHz, DMSO) δ 171.77, 153.91, 150.52, 142.41, 133.33, 129.31, 127.87, 127.26, 124.30, 121.94, 116.42, 112.40, 106.77, 36.17. MS(ESI): *m*/*z* [M]^+^ = calcd. for C_18_H_19_N_2_S^+^ 295.12; found 295.16.

### I-motif folding and ThT binding

Purified oligonucleotides were obtained as powders from Sangon Biotech (Shanghai, China), dissolved in TE buffer, and then quantified with UV–Vis spectroscopy. 1 μM i-motif DNAs in pH 5 TAE buffer containing 2 mM MgCl_2_ were incubated for 10 min at 90°C and then cooled slowly, allowing them to fold at room temperature. To the DNA solution, 3 μM ThT was added and kept at 22°C for over 5 min before fluorescence measurement.

### FNA assembly

The FNA device (the sequences were shown in Supplementary Table S1) consists of a smaller tetrahedron (Th46) and a bigger one (Th58), of which two vertexes are tethered with two component strands of the bimolecular TiM9. 1 μM Th46 and Th58 were separately prepared in the TE buffer and mixed at equivalent molar concentration, then underwent a heterodimeric assembly at 0.4 μM in pH 5 TAE buffer during an incubation period of 8 h at 37°C.

### Native PAGE

DNA nanoassemblies were analyzed with 6% native PAGE that ran under 96 V (6 V/cm) for 5 h. The gel was then stained in 1× GelRed solution and imaged by a Bio-Rad Gel Doc™ EZ imager (USA).

### CD measurements

400 μl of 50 μM i-motif DNAs in pH 5 TAE buffer containing 2 mM MgCl_2_ were measured in an optical chamber (1-mm path length) with a JASCO J-1500 spectropolarimeter (Tokyo, Japan). Dry purified nitrogen was used to maintain a deoxygenation atmosphere. The solution background was subtracted from the CD signal.

### Melting curves

400 μl of 50 μM DNA i-motifs in pH 5 TAE buffer containing 2 mM MgCl_2_ were denatured at a rate of 0.5°C/min and monitored at 290 nm with the J-1500 spectropolarimeter. Normalized CD signal was adopted.

### Fluorescence spectroscopy

The operation of the FNA device (0.4 μM) was monitored by the fluorescence of ThT (0.4 μM) in the TAE buffer (pH 5) containing 2 mM MgCl_2_. This FNA system was switched between pH 5 and pH 8 by alternate addition of 0.4% (v/v) 6 M HCl and 6 M NaOH, followed by a 30-min incubation at 60°C. It allows the i-motif DNA on the vertexes of two tetrahedrons to fold properly. The fluorescence spectra excited at 442 nm were recorded by a Hitachi F-4600 Fluorescence spectrometer (Tokyo, Japan). The buffer background was subtracted from the fluorescence spectra to reduce the light scattering influence especially in the case of measuring the weak fluorescence of ThT alone.

### MDS calculation

The model of TiM9 was built on the basis of an already known i-motif structure (PDB ID: 1YBL) from Protein Data Bank,59 then minimized and equilibrated by MDS using the Gromacs-5.1.4 suite with AMBER99SB force field60 under the solvents condition mimicking the experiments. H^+^ and Cl^−^ were added as counter ions, then the system was solvated in TIP3P water molecules, which were extended up to 8.0 × 8.0 × 14.0 Å in a cubic box. Totally 6 ns of MDS in npt ensemble was run for the solvated system and the last 3 ns MDS was used as product, with coordinates saved for every 10 ps. The ground state of ThT was optimized by quantum chemical calculations using the Gaussian 16 program. Becke's three-parameter hybrid exchange function with the Lee-Yang-Parr gradient-corrected correlation functional (B3LYP functional) was used and 6-31G(d) basis set was chosen. The molecular interactions of TiM9 with ThT were studied by software AutoDock 4.2.6 with the Lamarckian genetic algorithm61. The grids number were 110 × 110 × 110 and a grid spacing was 0.375 Å.

### AFM characterization

The monomeric and dimeric DNA tetrahedrons were prepared with a total concentration of 100 nM. The prepared samples were scanned in scanasyst-air mode by a Multimode 8 Atomic Force Microscope (Bruker Inc.).

### Cell culture and confocal fluorescence imaging

HepG2 cells were cultured in DMEM medium supplemented with 10% (v/v) FBS and 2% (v/v) 100× stock solution of penicillin-streptomycin mixture. 1 μM LysoTracker Red DND-99 was first added to HepG2 cell culture medium and incubated at 37°C for 20 min. After removing the LysoTracker dye by changing fresh cell culture medium, a mixture of cholesterolated Th46 and Th58 (each 1 μM) in pH 5 TAE buffer with 2 mM MgCl_2_ was added and incubated for 1 h. Finally, 0.4 μM ThT was added and incubated for 5 min for fluorescence imaging. In the control experiments at pH 8, however, the ThT dye needed to be removed before cell imaging for an appropriate background. The fluorescence signals of ThT and LysoTracker were collected with the excitation wavelength of 405 and 590 nm, respectively. All the micrographs were taken on a confocal laser-scanning microscope (Zeiss, Model LSM 880).

## RESULTS

### Design and ligand-binding behaviors of the i-motif/miniduplex hybrids

**Figure 2.**
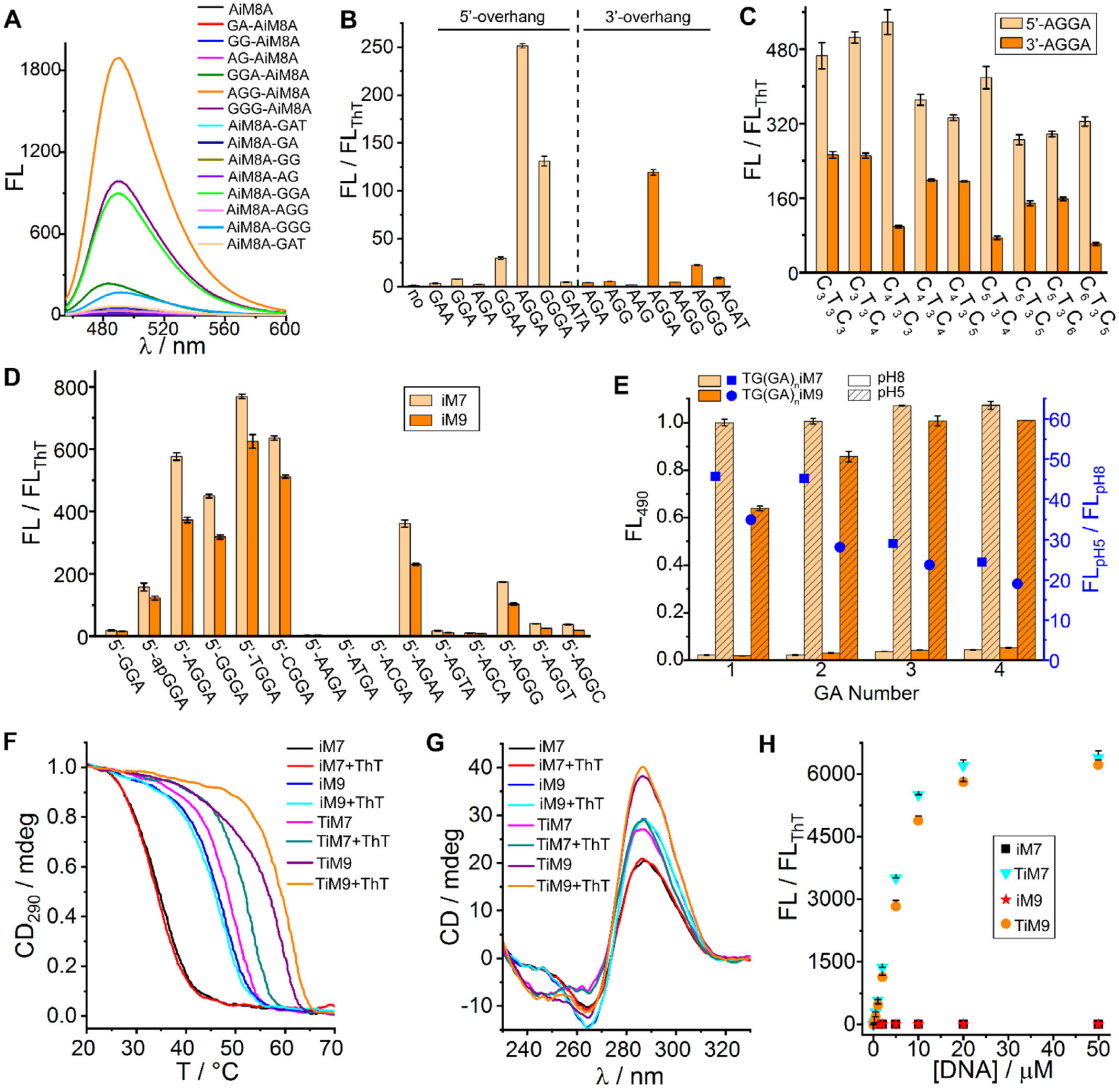
Identification of the ThT-interactive i-motif hybrid structures (each at 1 μM) in TAE buffer at pH 5 with 2 mM MgCl_2_ in the presence of 3 μM ThT. (**A**) Fluorescence spectra of ThT incubated with the bimolecular i-motif AC_4_T_3_C_4_A (AiM8A) containing different 5′- or 3′-overhangs. (**B**) Distinct behaviors of various 5′-/3′-overhangs when enhancing the fluorescence of ThT. (**C**) General fluorescence improvement effect of the 5′-/3′-AGGA overhangs on different bimolecular i-motifs. (**D**) Addressing the ThT-interactive preference site via one-by-one base substitution for the AGGA overhang of C_4_T_3_C_3_ (iM7) and C_5_T_3_C_4_ (iM9). (**E**) Effect of the 5′-overhang length on the ThT fluorescence that was monitored at 490 nm (FL_490_) and normalized. (**F**, **G**) Melting curves and CD spectra of 50 μM iM7, iM9, and their hybrids (TiM7 and TiM9) with the 5′-TGGA overhang in the absence and presence of 150 μM ThT. (**H**) Fluorescence titration of 3 μM ThT with different concentrations of i-motifs and hybrids.

As a prelude to engineering the i-motif/miniduplex hybrids, we first sought an appropriate appendix. Given the effect of flanking nucleotides on the i-motif stability (Supplementary Figure S3) (40), we began with an ideal i-motif AC_4_T_3_C_4_A (AiM8A) to explore the ThT-interactive structures. Several nucleotide partners favorable for ThT binding in psDNAs (24) are here chosen and appended to the 5′ or 3′ terminal of AiM8A, respectively. When incubated with ThT, the resulting i-motifs display distinct behaviors in fluorescence improvement at pH 5 (Figure 2A) whereas no obvious difference was observed at pH 8 (Supplementary Figure S4), indicating a pH dependence that is a characteristic of i-motif interactions with ligands (16,41). However, the big difference shown in Figure 2A reveals that the flanking nucleotides apparently play an important role in the ThT binding. Comparing these appendices combined with the terminal adenine of AiM8A, we notice that a tetranucleotide AGGA behaves as a favorable overhang for ThT binding on the both ends (Figure 2B). This tetranucleotide is then appended to the 5′ or 3′ terminal of the reported bimolecular i-motifs (30). The resulting i-motifs, especially those with 5′-overhang, are all found to dramatically enhance the ThT fluorescence (Figure 2C), comparable to the enhancement effect of most G4s (22). A similar phenomenon is also observed when tetramolecular and unimolecular i-motifs are tested (Supplementary Figure S5). Since the G and A nucleotides can form G•G and A•A homo base pairs stabilizing pdDNAs (42) and benefiting to ThT binding (24,25), the above phenomenon strongly suggests that the two AGGA overhangs on i-motifs tend to form a short pdDNA, and thereby extend the core structure. The possibility of unpaired overhangs is rationally excluded according to two facts: namely that the overhangs cannot function at basic pH (Supplementary Figure S4) and unimolecular i-motifs do not work with only one appendix (Supplementary Figure S5A).

It's observed that the 5′-overhang generally behaves better than the 3′-overhang especially on C_4_T_3_C_3_ (iM7) and iM9 (Figure 2C). Hence, these two i-motifs with 5′-overhang are chosen to address the preference site for ThT binding *via* one-by-one replacing AGGA with other nucleotides. We notice that there is no longer an improvement on the ThT fluorescence when the second nucleotide is displaced (Figure 2D), underlining this site as a critical domain in the overhang for ThT binding. In contrast, the first nucleotide can be replaced with any analogue (e.g. unpaired thymine) and even an apurinic (ap) acid (43), but the loss of it (i.e. shortening the overhang to GGA) makes the structures unable to effectively combine with ThT. The remaining GA motif thus seems like a stabilizer for the overhang as it did in common pdDNAs (24,25), because the moderate ThT fluorescence still remains if it's replaced with an AA or GG motif. Taken together, these data evidence a preferential binding site for ThT near the G•G base pair at the second site of the parallel-paired overhangs on bimolecular i-motifs. This is well supported by molecular dynamics simulations that propose a molecular docking model for ThT binding in the i-motif/miniduplex hybrid (Figure 1C), just like in parallel homoduplexes (25). The GGA motif in this overhang is mainly being made to an ultrashort non-canonical miniduplex. For more details, see Supplementary Figure S6 in Supporting Information.

Among all tested 5′-overhangs, TGGA is proven the best candidate on both iM7 and iM9 (Figure 2D). Extending this overhang simultaneously increases the ThT fluorescence at acidic and alkaline pHs, causing a drop in the signal-to-background for pH switch (Figure 2E). Hence, we concentrated on iM7 and iM9 appended with 5′-TGGA (hereafter named TiM7 and TiM9) and further tested them by thermal denaturation, circular dichroism (CD), and fluorescence titration. Figure 2F shows that both TiM7 and TiM9 display an improved melting temperature (*T*_m_) even in the absence of ThT as compared to iM7 and iM9, accompanied by an increase in the CD signal (Figure 2G). These results show a general positive effect of the 5′-TGGA overhang on the thermal stability of i-motifs, consistent with the previous report (40) and our observations from Supplementary Figure S3. Upon addition of ThT, the thermal stability of TiM7 and TiM9 is obviously further improved, whereas that of iM7 and iM9 is unchanged (Figure 2F). It demonstrates that the ligand ThT can stabilize the i-motif hybrids TiM7 and TiM9 rather than the conventional i-motifs, unlike some i-motif-interactive ligands (44,45). At the saturated binding state, TiM7 and TiM9 can enhance the ThT fluorescence by over 6000-fold (Figure 2H), exceeding any of previously reported DNA forms including G4s (22,33) and pdDNAs (24,25). Such an unprecedented enhancement is mainly attributed to not only the extremely low background fluorescence of ThT but also the ultrahigh binding efficiencies of the i-motif hybrids. The latter is further confirmed by a parallel comparison between different DNA structures under the same conditions (Supplementary Figure S7). In addition, both TiM7 and TiM9 can bind ThT with *K*_d_ ∼ 3.9 and 5.7 μM, respectively (Supplementary Figure S8). It's comparable to that of a well-known DNA aptamer (46), which binds adenosine or ATP with *K*_d_ ∼ 6 μM. This comparison underlines a high affinity of our designed i-motif hybrids for ThT binding.

**Figure 3.**
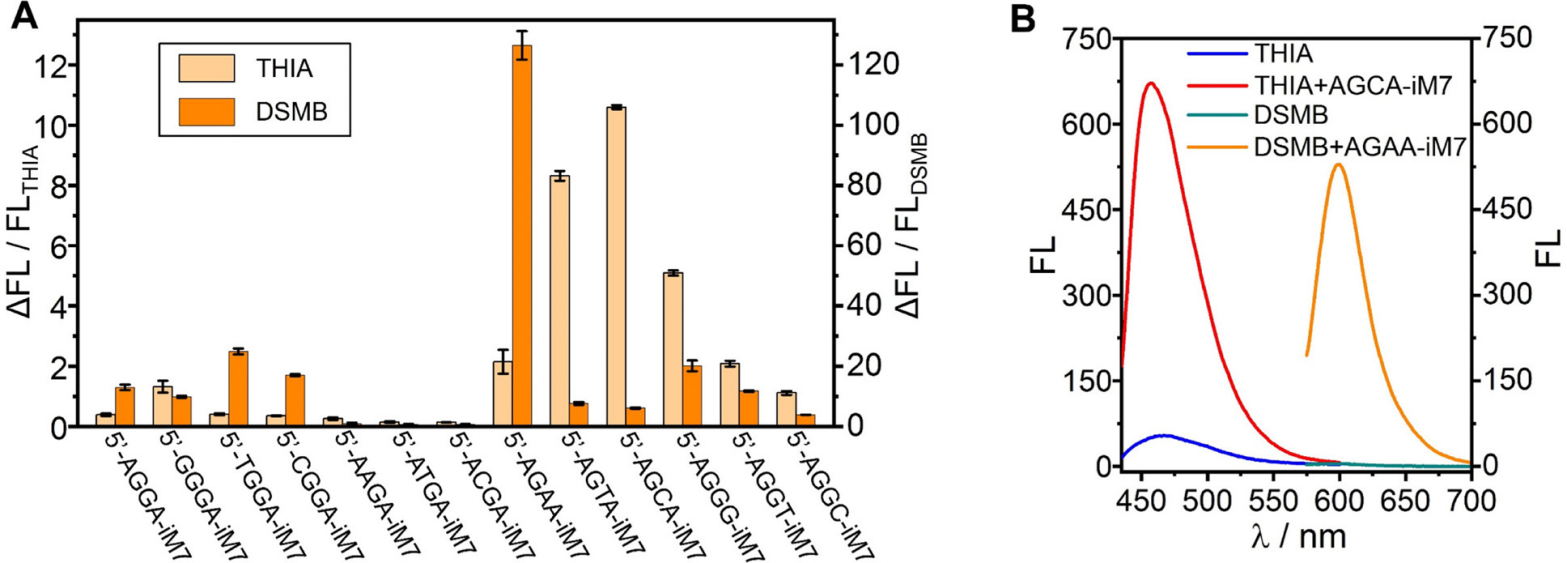
Identification of the i-motif/miniduplex hybrids (1 μM) interacting with other molecular motors (3 μM) in TAE buffer at pH 5 with 2 mM MgCl_2_. (**A**) Effect of different 5′-overhangs of iM7 on the fluorescence of THIA and DSMB monitored at 467 and 608 nm, respectively. It shows a dependence of the overhang sequence on the ligand structures. (**B**) Fluorescence spectra of THIA and DSMB excited at 420 and 555 nm, respectively, in the absence and presence of the hybrids.

To further demonstrate the generality of our design strategy for ligand-interactive i-motif/miniduplex hybrids, two other benzothiazole dyes THIA (47) and DSMB (39) are tested in place of ThT. Figure 3A shows that the best tetranucleotide overhang varies with different molecular structures of target ligands. For example, the AGCA 5′-overhang displays the optimal performance for binding THIA, whereas AGAA is the best motif for DSMB. However, for both THIA and DSMB, the second nucleotide in the overhang also plays a crucial role in ligand binding to the hybrid structures, just like in the TiM9/ThT complex. It underlines a generic rule in the interactions between the i-motif/miniduplex hybrids and different benzothiazole molecular rotors.

Compared with ThT and DSMB, THIA is found to display a much lower fluorescence enhancement after being bound by the optimal i-motif/miniduplex hybrid, mainly attributed to its higher fluorescence in the unbound state (Figure 3B). Among these molecular rotors, the fluorescence of ThT can be enhanced to the maximum by the optimal hybrids under the identical conditions. It can in principle provide a high signal-to-background ratio when utilizing the designed hybrids (TiM7 and TiM9) together with ThT as a novel light-up system for monitoring DNA assembly and bioimaging.

### Utilizing the hybrids for label-free FNA assembly and cell imaging

**Figure 4.**
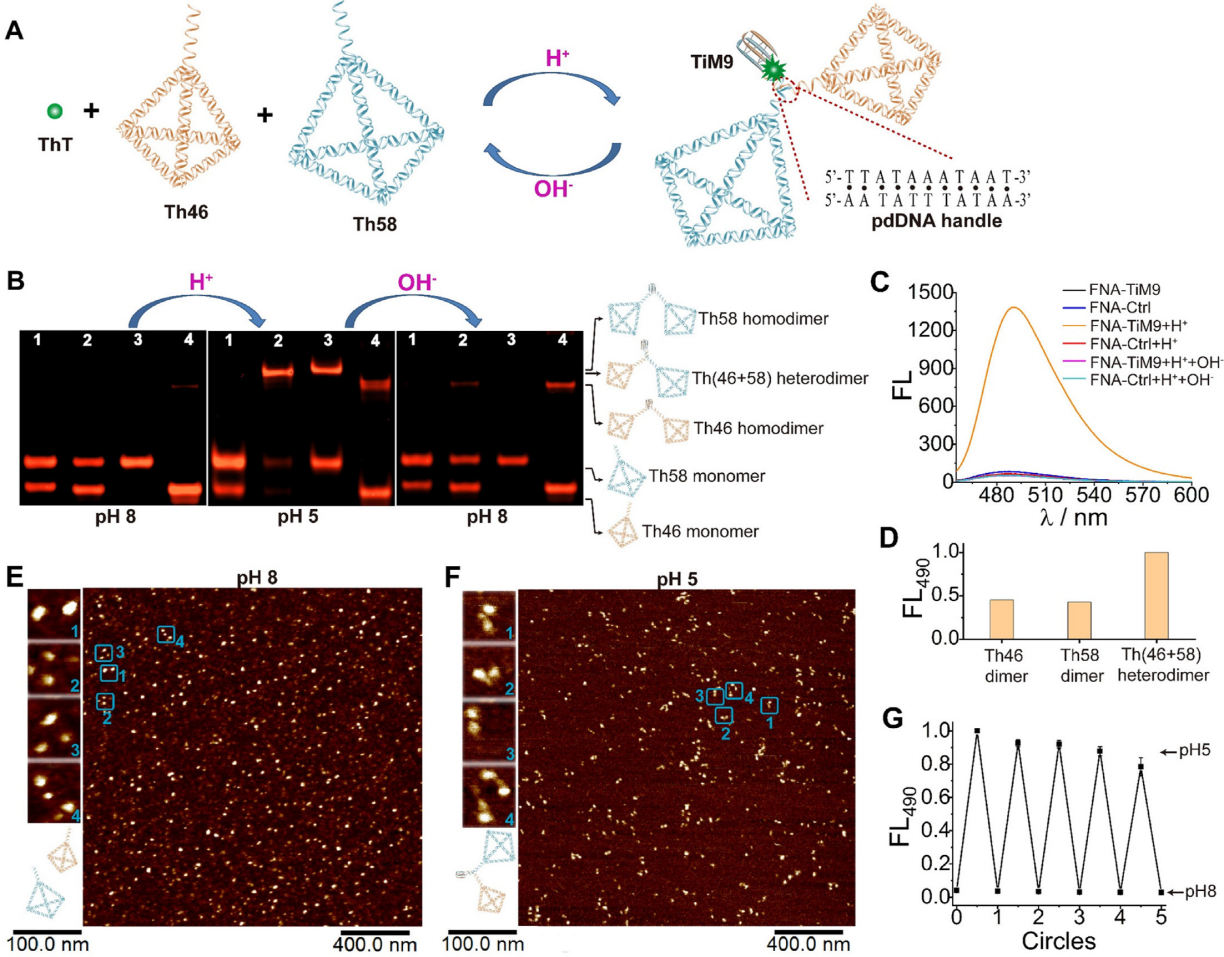
Characterization of the pH-switched FNA device. (**A**) Schematic for the dimeric assembly of FNA-TiM9 in response to pH change. (**B**) Native PAGE (6%) for analyzing pH-controlled assembly of 0.2 μM tetrahedrons (total concentration). Lanes: 1, FNA-Ctrl; 2, FNA-TiM9; 3, Th58-TiM9; 4, Th46-TiM9. (**C**) Fluorescence spectra of 0.4 μM ThT plus FNA-TiM9 or FNA-Ctrl in response to pH change. (**D**) Fluorescence comparison between 0.2 μM heterodimer and homodimers of Th46 and Th58. (**E** and **F**) AFM images of the FNA device at pH 8 and 5. Left panels show amplified pictures of monomeric and dimeric tetrahedrons, and the corresponding entire images are provided as Supplementary Figure S10. (**G**) Working circles of the FNA device switched between pH 8 and 5.

As TiM9 is more stable than TiM7 (Figure 2F), the former behaves better than the latter when tethered to two tetrahedrons (Th46 and Th58), a common type of FNA (34–36). So, TiM9 is here adopted to functionalize this FNA system (FNA-TiM9) through a pdDNA handle that promotes the heterodimerization of Th46 and Th58 (Figure 4A). In the heterodimerization process, this handle can form a parallel duplex, and thereby improving the stability of the dimeric nanostructure. In contrast, it just behaves as a DNA linker in the homodimer. For this reason, a heterodimeric nanostructure is more stable than a homodimer, which contributes to a higher efficiency for the heterodimer formation. For more details, see Supplementary Figure S9 in Supporting Information.

FNA-TiM9 was then analyzed by native polyacrylamide gel electrophoresis (PAGE) to show its pH-induced dimeric assembly (Figure 4B, lane 2). As the control, a FNA system without TiM9 (FNA-Ctrl) is investigated together (lane 1). At pH 8, there are only two bands of monomeric tetrahedrons in lane 2, indicating no dimer formation induced by the pdDNA handle. Upon lowering pH to 5 (Figure 4B, middle panel), a slowly moving band emerges in lane 2 and meanwhile those of monomers almost disappear, demonstrating that most tetrahedrons are dimerized due to the i-motif folding (18). This dimer displays a middle mobility between those of Th58 and Th46 self-dimers (lanes 3 and 4), consistent with the electrophoretic feature of a heterodimer (30). The pH-induced tetrahedron dimerization is accompanied by an over 20-fold increase in the ThT fluorescence (Figure 4C), attributed to the aforementioned interaction between ThT and folded TiM9. Such a big fluorescence change allows TiM9 to easily monitor the response of FNA device to pH change. We notice that the self-dimerization of Th58 or Th46 alone is not complete under identical conditions, reflected by a large amount of remaining monomers (Figure 4B, middle panel, lanes 3 and 4) and further confirmed by fluorescence spectroscopy (Figure 4D). At the same concentration of total tetrahedrons, the heterodimer displays an over 2-fold higher fluorescence than Th46 and Th58 self-dimers. This difference between the tetrahedron heterodimer and homodimers ensures the controllability of DNA assembly that is usually a prerequisite for *in vitro* and *in vivo* building switchable DNA nanosensors (18,30).

Since dimeric DNA nanostructures can be visualized by atomic force microscopy (AFM) (8,9,18,35), we employed it here to further verify the pH-induced heterodimerization of Th46 and Th58. Figure 4E shows many bright dots with a good monodispersity at pH 8, consistent with the AFM characteristic of monomeric tetrahedrons (18,48). Among these dots, different sizes are obviously observed especially in the amplified figures (Figure 4E, left panels), corresponding to two different tetrahedrons (i.e. Th46 and Th58) with 14-bp and 18-bp edge length, respectively. Under acidic conditions (pH 5), there are many pairs of bright dots closely presented in the AFM image (Figure 4F). Amplifying some of them shows a clear dimeric structure of two tetrahedrons (Figure 4F, left panels), in which one tetrahedron is a little smaller than another and the DNA conjugate between two tetrahedrons is also clearly observed in some cases (panels 1 and 2). Undoubtedly, Th46 and Th58 undergo a controllable heterodimerization induced by the folding of TiM9 at acidic pH, consistent with PAGE and fluorescence results.

We further tested the reconfigurability of the aforementioned FNA assembly *via* resetting pH to 8. Figure 4B (right panel) shows that two bands of monomeric Th46 and Th58 appear again, and meanwhile that of the dimer becomes almost unobservable (lane 2). This is accompanied by a sharp decrease in the ThT fluorescence that returns to the initial state (Figure 4C). These observations apparently demonstrate that our FNA device is reconfigurable in terms of structural intactness and fluorescence readout. It can be cycled back and forth between monomeric and dimeric states many times (Figure 4G), in a fully reversible fashion like its fluorescent-labelled counterparts (9,18,20,29,30).

**Figure 5.**
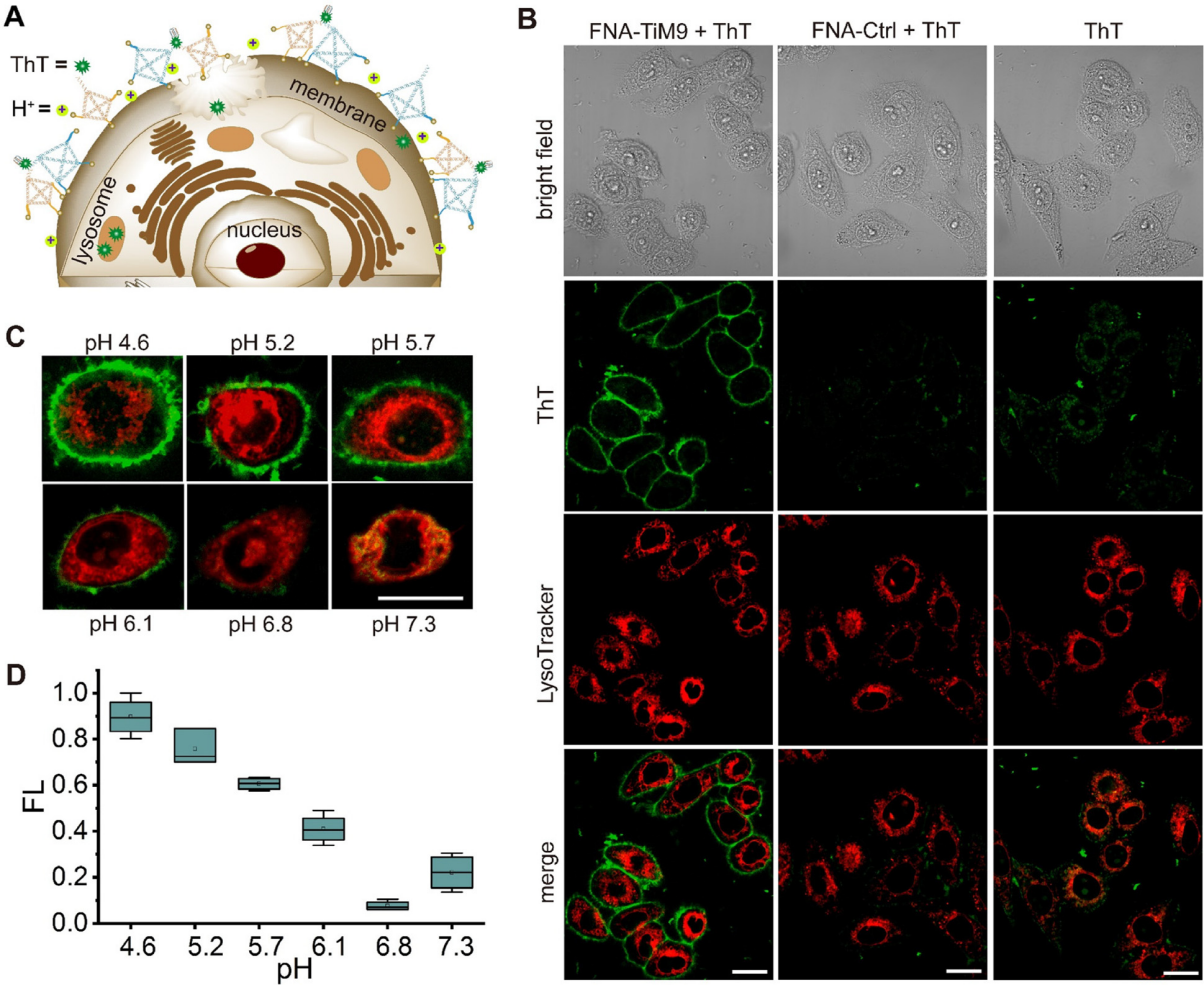
Label-free cellular membrane imaging with the FNA device. (**A**) Dynamic assembly of cholesterol-anchored FNA-TiM9 in acidic cellular membrane microenvironments. (**B**) Confocal fluorescence images of HepG2 cells treated with 0.4 μM FNA-TiM9 or FNA-Ctrl at pH 5.2 for 1 h, followed by 5-min incubation with 0.4 μM ThT. (**C**) Fluorescence imaging in response to different extracellular pHs. The scale bar in each case is 20 μm. (**D**) Quantitative analysis of fluorescence intensity on the cell membrane with ImageJ.

Given that i-motif-modulated DNA nanodevices are particularly favored in acidic cellular environments (18–20), we next sought to manipulate the FNA-TiM9 system at acidic cancer cell surfaces (49). Here the FNA-TiM9 system was *in situ* operated on the membrane surfaces of HepG2 cells, as depicted in Figure 5A. It's known that tetrahedrons can easily enter cells *via* endocytotic internalization (48,50), i.e. they do not remain on cell membrane surfaces over long periods of time. To overcome this issue, a cholesterol anchor widely used on cell membranes (20,37) is tethered to three vertexes of Th46 and Th58. This allows the two tetrahedrons to remain on the cell surfaces for a period of over one hour, enabling *in situ* assembly into the ThT-interactive heterodimeric structure at acidic extracellular pH.

Figure 5B shows that after treatment with FNA-TiM9, the membranes of cells display bright fluorescence from ThT, which is not observed in the cells treated with FNA-Ctrl. Comparing with the bright-field image, we notice that the ThT fluorescence primarily appears on the cell surface. For localizing tetrahedrons, a lysosome dye (LysoTracker RED DND-99) (51,52) is incubated with cells before they are treated with tetrahedrons. Merging the ThT channel with LysoTracker shows that tetrahedrons are not transported into lysosomes, which are their final destinations during normal cell endocytosis (18,48). This validates the stable attachment of the FNA-TiM9 system on the cell surfaces *via* cholesterol anchoring to the cell membrane (20,37). Upon adjusting the extracellular pH to 8, the ThT fluorescence on the cell surface disappears entirely (Supplementary Figure S11), indicating no dimerization of the FNA device. It demonstrates that the designed i-motif/miniduplex hybrid functions well in real cellular environments, acting as both the light-up and modulating systems for pH-switched FNA nanoassemblies and label-free cell membrane imaging. Further, we notice that the fluorescence brightness on cell surfaces is highly dependent on extracellular pH value (Figure 5C). This is more straightforward when quantitatively analyzing the fluorescence intensity on the cell membrane within a larger field of view (Supplementary Figure S12) using the software tool ImageJ (19,53), indicating that the fluorescence brightness almost linearly decreases as extracellular pH increases from 4.6 to 7.3 (Figure 5D). In this sense, the FNA-TiM9 system behaves like a novel label-free fluorescent sensor in response to extracellular pH changes, as its fluorescent-labelled counterparts did (20,37,38,54,55).

## DISCUSSION

A number of fluorescent dyes were reported to combine with the i-motif structures. Most of them (e.g. ThT) display an enhanced fluorescence emission when bound to the folded i-motifs (16,17,41,56), although it is much lower than the fluorescence enhancement by G-quadruplexes under the same conditions (Supplementary Figure S1A). However, a few reports showed that some dyes are quenched by the folded i-motifs. Recently, i-motif DNAs were found to have the highest quenching on the fluorescence of labelled FAM as compared to other DNAs, which was attributed to the interaction between FAM and i-motifs that may result in a nonfluorescent species (57). Such a mechanism of quenching is distinct from the enhancement effect of our i-motif hybrids on the ThT fluorescence. As we know, ThT has two aromatic rings that can rotate freely, and it has a extremely low fluorescence in the unbound state due to intramolecular charge transfer (21). When bound to any form of DNAs (22,24,25,31–33), its two aromatic rings are restricted in the excited state (58), generally accompanied by a sharp increase in the fluorescence emission.

From Figure 2C it's found that the overhang AGGA always works better for ThT binding at the 5′-terminal than at the 3′-terminal of all tested i-motifs. According to the experimental data (Figure 2D), the second nucleotide of the motif NGGA (N = T, C, A, G) plays a crucial role in the ThT binding, which is thought to govern the interaction of TiM9 with the rotatory dimethylaminobenzene ring of ThT in the MDS model (Figure 1C). When appended at the 5′-terminal, this binding site is relatively far from the i-motif core structure as compared to that at the 3′-terminal. A smaller steric hindrance may account for a relatively high performance of the 5′-overhang for ThT binding. Nevertheless, we also tried to design certain i-motif sequences with the 3′-overhang that can work well. According to our observations from Figure 2C, we chose the two i-motifs C_3_T_3_C_4_ and C_4_T_3_C_5_ for the 3′-overhang optimization, and demonstrated that 3′-GGGA works quite well (Supplementary Figure S13), with a high enhancement on the ThT fluorescence comparable to that of 5′-TGGA appended to C_4_T_3_C_3_ and C_5_T_3_C_4_ (Figure 2D).

We notice that the sequences of optimal overhangs vary with the ligands, as shown in Figures 2D and 3A. For example, TGGA is the best overhang for ThT binding. The MDS results show the T bases are not involved in the formation of parallel miniduplex (Figure 1C and Supplementary Figure S6), which can rationally account for why the replacement of T by other bases does not dramatically influence on the binding efficiency (Figure 2D). That is, 5′-NGGA (N = T, C, A, G) is proven the optimal overhang on i-motif hybrids in terms of ThT binding. Likewise, further experiments demonstrate that 5′-NGAA (N = T, C, A, G) is the optimal overhang for DSMB binding (Supplementary Figure S14). Such a similarity implies that the first base in this 5′-overhang does not participate in the miniduplex formed by the GAA motif, just like the MDS model of TiM9. However, the sequence difference between 5′-NGGA and 5′-NGAA at the third site will influence the distance between G•G and A•A homobase pairs in the miniduplex (Supplementary Figure S6), which may account for their selectivity for different fluorescent ligands.

## CONCLUSION

We have demonstrated a conceptually new strategy for general engineering of i-motif/miniduplex hybrids with high capacity for binding ThT and other molecular rotors. It allows the hybrids to function as a high signal-to-background light-up system as well as a modulating unit for a pH-driven dimeric FNA nanoswitch that can be manipulated *in situ* on cellular membrane surface. This lays the foundation for label-free fluorescence bioimaging in response to extracellular pH changes.

It's known that functional biomolecular dimerization plays a crucial role in many physiological processes (59–67). Some of them take place on cell membrane surfaces to regulate signal transduction (67) and gene expression (68). For this reason, there is an emerging interest in DNA dimeric regulation on cell surfaces for different purposes (69–73). When tethered to dimeric and multimeric DNA aptamers that specifically bind cellular membrane proteins (69–72,74,75), our designed hybrids will provide a facile and useful tool for light up and meanwhile modulate target recognition and DNA computation on cell surfaces (76,77).

## Supplementary Material

gkaa020_Supplemental_File
