# Supplementary material for: I-Motif/miniduplex hybrid structures bind benzothiazole dyes with unprecedented efficiencies: a generic light-up system for label-free DNA nanoassemblies and bioimaging
Source: Nucleic Acids Res. 2020 Jan 17;48(4):1681–90. doi: 10.1093/nar/gkaa020 (PMC7039006; doi:10.1093/nar/gkaa020)
Supplement: gkaa020_Supplemental_File [file gkaa020_supplemental_file.pdf]

# Supporting Information

## I-Motif/Miniduplex Hybrid Structures Bind Benzothiazole Dyes with Unprecedented Efficiencies: A Generic Light-Up System for Label-Free DNA Nanoassemblies and Bioimaging

Lili Shi†, Pai Peng†, Jiao Zheng, Qiwei Wang, Zhijin Tian, Huihui Wang and Tao Li*

Department of Chemistry, University of Science and Technology of China, 96 Jinzhai Road, Hefei, Anhui 230026, China.

† These authors contributed equally to this work.

* To whom correspondence should be addressed. Tel: +86 551 63601813; Email: tlitao@ustc.edu.cn

## Supplementary Figures and Discussions

G-quadruplexes (G4s) and i-motifs share a common feature that they both have the ability to binding many fluorescent ligands (1-6), such as the molecular rotor thioflavin T (ThT) (6). Free ThT has very week fluorescence due to twisted internal charge-transfer (7), while it displays a dramatically enhanced fluorescence when the intramolecular rotation is restricted by the interactions with DNAs (3). Importantly, ThT is able to bind both G4s and i-motifs, and hence chosen to make a brief comparison between these two kinds of quadruplex DNAs for ligand binding. Figure S1 shows that under the cell-mimicking acidic ionic conditions, different versions of human telomeric i-motifs display much weaker improvement on the ThT fluorescence than human telomeric G-quadruplexes. In this case, ThT is unlikely to monitor the formation of i-motif structures in the context of large DNA nanoscaffolds. It may account for why i-motifs are rarely used together with their ligands as the light-up systems for DNA sensing and assembling, not as G4s did (7-10).

**A**

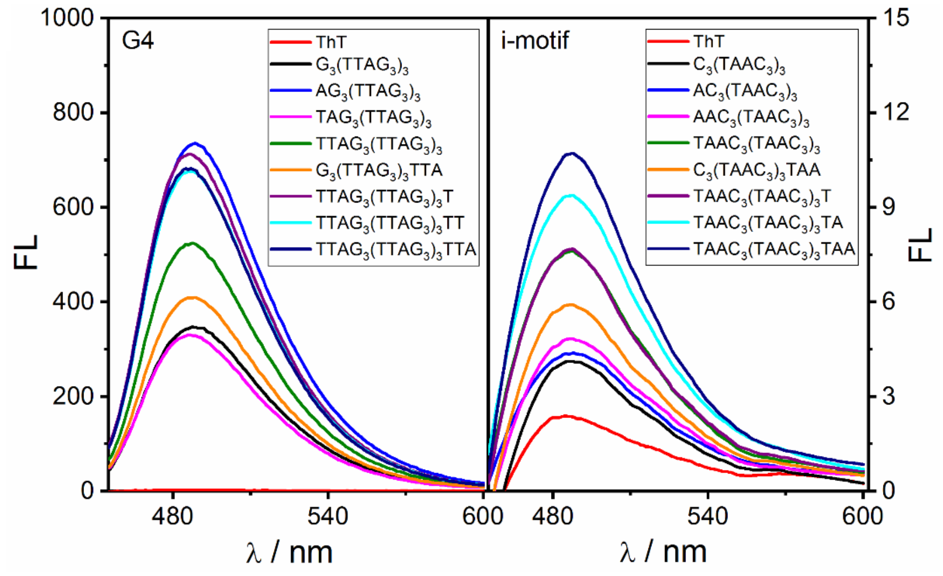

**B**

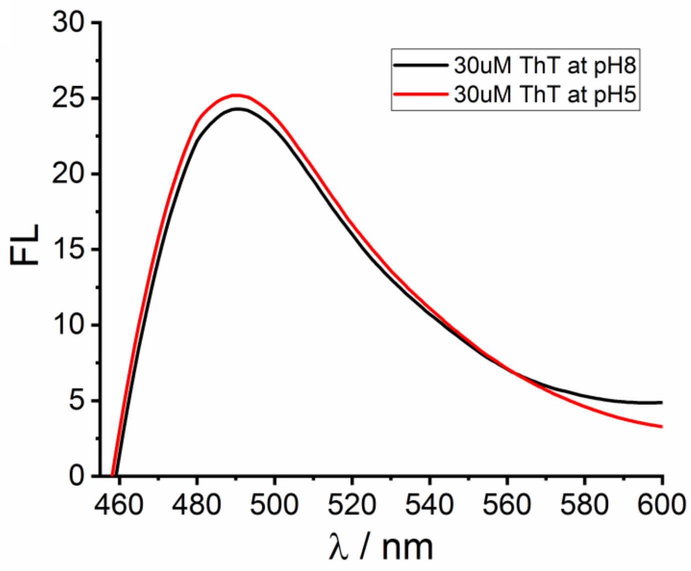


**Figure S1.** (A) Fluorescence spectra of 3 μM ThT in the absence and presence of 1 μM DNAs (different versions of human telomeric G-quadruplexes (G4) and i-motifs) under the cell-mimicking acidic ionic conditions (pH 5.0, 2 mM $MgCl_2$ and 60 mM KCl). (B) pH effect on the fluorescence of ThT itself. No obvious change is observed during the pH-switching process.

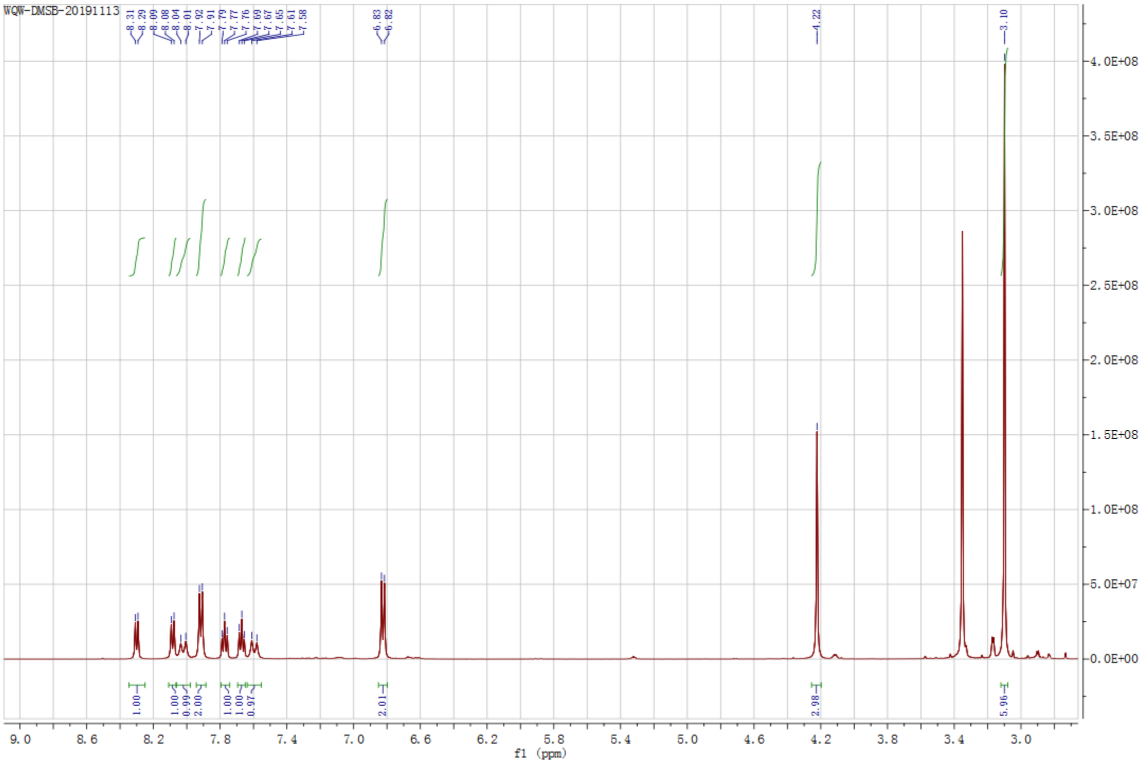
WQW-DMSB-20191113
8.31
8.29
8.09
8.08
8.04
8.01
7.92
7.91
7.79
7.77
7.76
7.69
7.67
7.65
7.61
7.58
6.83
6.82
4.22
3.10
1.00
1.00
0.99
2.00
1.00
1.00
0.97
2.01
2.98
5.96
f1 (ppm)

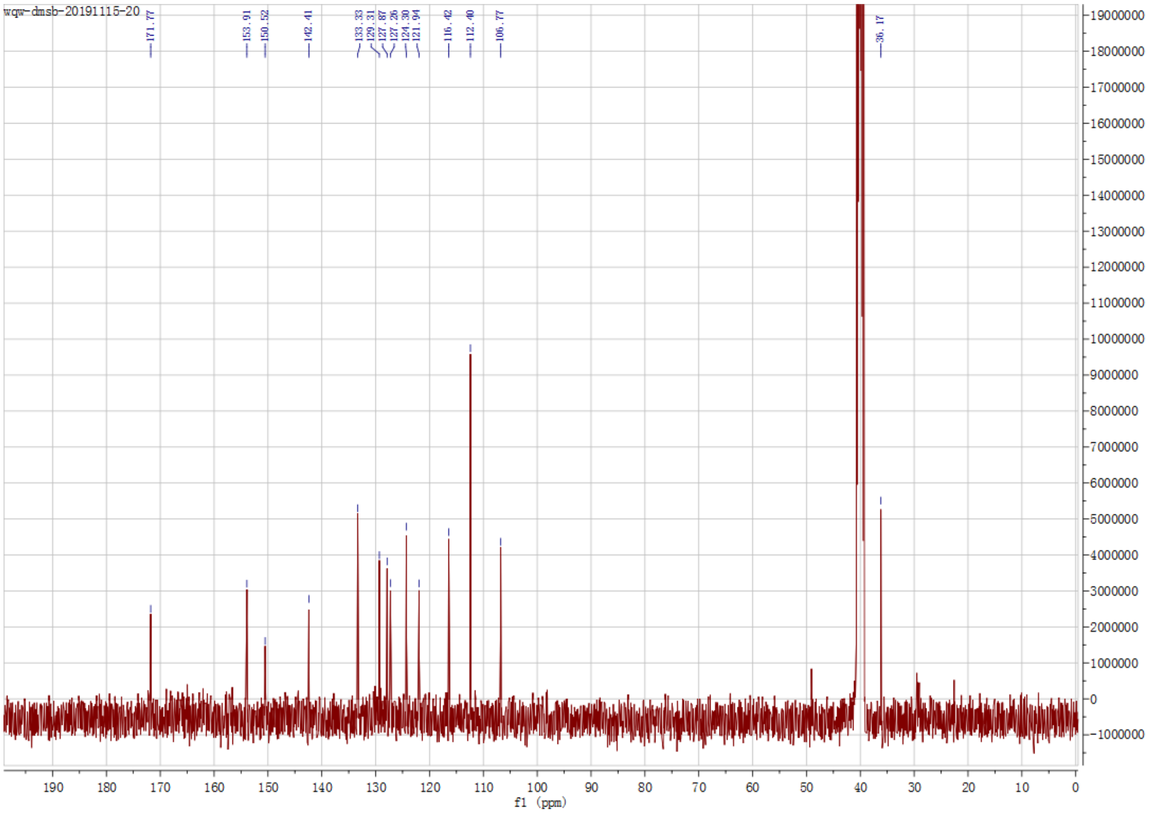
wqw-dmsb-20191115-20
171.77
153.91
150.52
142.41
133.33
129.31
127.87
127.26
124.30
121.94
116.42
112.40
106.77
36.17
f1 (ppm)

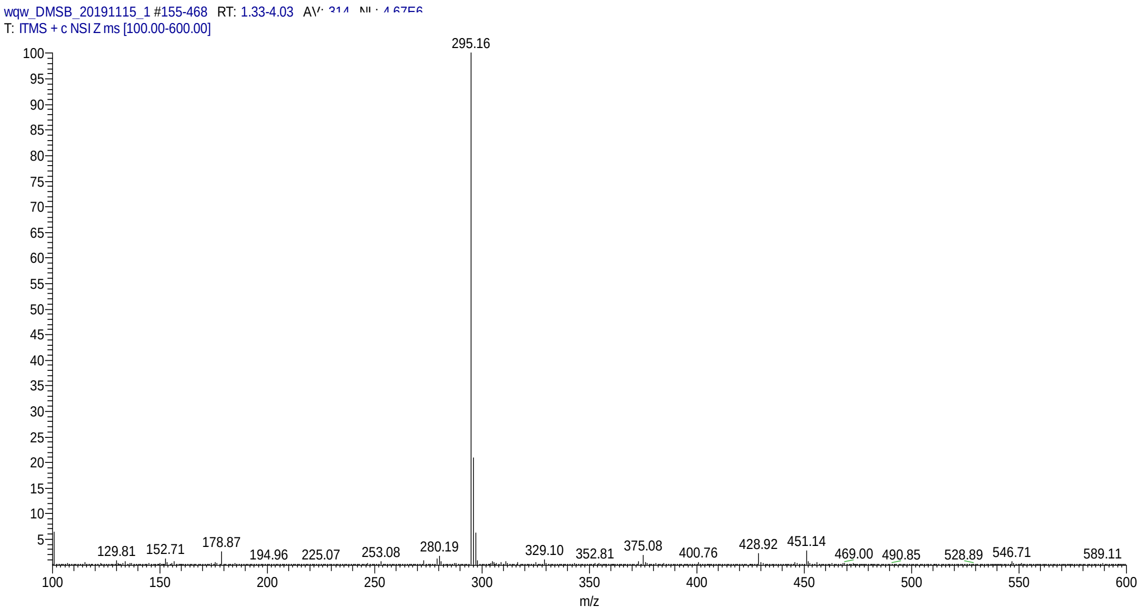


**Figure S2.** $^{1}$HNMR (top), $^{13}$CNMR (middle) and ESI-MS (bottom) spectra of HPLC-purified DSMB.

For engineering the desirable i-motif hybrids, we firstly sought to figure out how the flanking nucleobases influence the i-motif structures, as the potential effect of flanking nucleotides on the i-motif stability has been reported previously (11). We append different nucleobases to the 5' termini of a previously used bimolecular i-motif $C_4T_3C_4$ (iM8) (12). Figure S3A shows that the most adjacent nucleobase especially adenine can obviously improve the thermal stability of the i-motif, mainly attributed to the stacking of nucleobases onto the C-C$^+$ base pair (11). Similar phenomena are also observed when flanking nucleobases onto the 3' termini (Figure S3B). As the influence of the second and third bases becomes much smaller (Figure S3A), the best stable $AC_4T_3C_4A$ (AiM8A) is regarded as an ideal candidate and chosen for i-motif engineering.

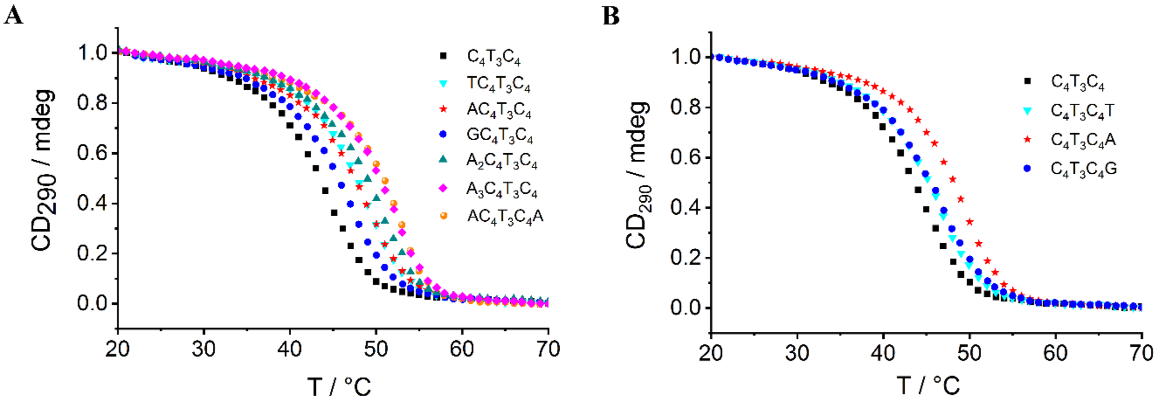


**Figure S3.** Effect of 5'-flanking (A) or 3'-flanking (B) nucleobases on the stability of bimolecular i-motif structures.

Then, several nucleotide partners favorable for ThT interactions with pdDNAs (13) are appended to the 5’ or 3’ terminal of AiM8A, respectively. The resulting i-motifs display distinct ability to combine with ThT, reflected by a big difference in the fluorescence of ThT at pH 5 (Figure S3A) whereas no obvious change at pH 8 (Figure S3B). This pH-dependent feature is usually observed in i-motifs interacting with ligands.(6,14) By comparing these tested overhangs of iM8, we find that a tetranucleotide AGGA on both ends favors the interaction of i-motifs with ThT (Figure S3C), and choose it for engineering the i-motif-based light-up systems.

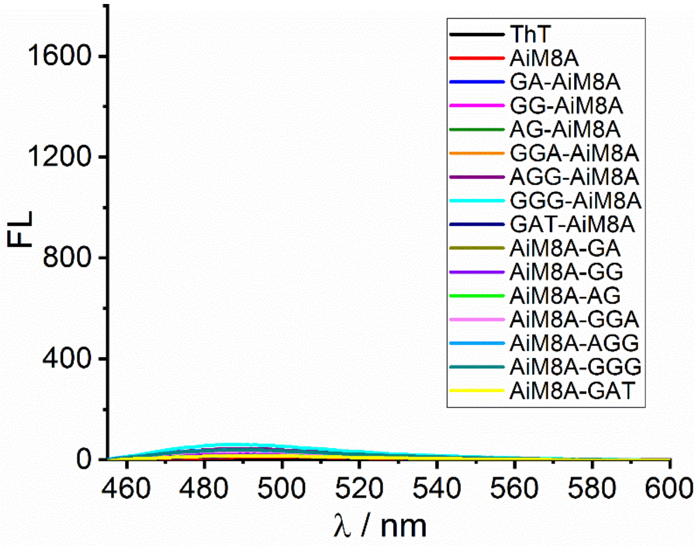


**Figure S4.** Fluorescence spectra of ThT incubated with AiM8A containing different 5’- or 3’-overhangs at pH 8.

Figure S4A shows that the 5'-overhang AGGA on tetramolecular i-motifs (C6 and C7) can sharply enhance the fluorescence of ThT, as there actually are four AGGA motifs in their tetramolecular structures (Figure S4B). For the unimolecular i-motifs (HTC21 and TTC21), however, there is only one AGGA motif on the 5' terminal of their folded structures. In this case, no remarkable enhancement on the ThT fluorescence is observed, as the i-motif core cannot be extended by single tetranucleotide. Hence, another AGGA motif need to be added into the second loops of these unimolecular i-motifs (Figure S4C), allowing the two supplemental motifs to be paired and therefore provide a ThT-interactive preference site just as the overhangs on bimolecular or tetramolecular i-motifs do. As a result, remarkably enhanced fluorescence of ThT is observed (Figure S4A).

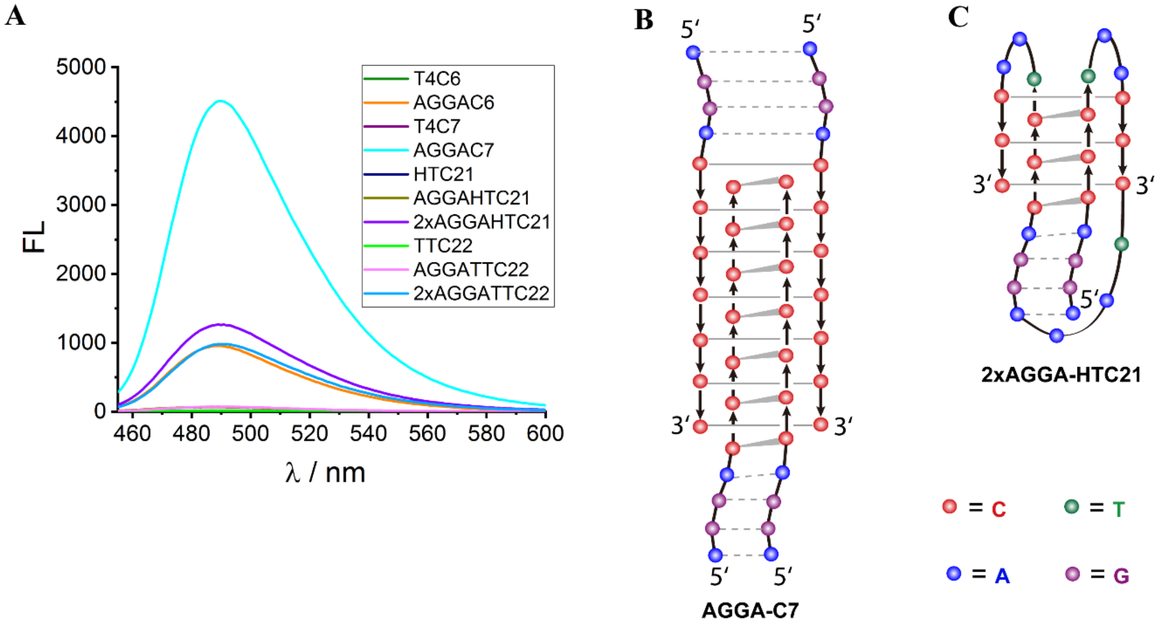


**Figure S5.** (A) Fluorescence spectra of ThT incubated with tetramolecular and monomolecular i-motifs with and without the 5'-AGGA overhang. (B) and (C) The proposed folded structures of AGGA-C7 (AGGACCCCCCC) and 2xAGGA-HTC21 (AGGACCCTAACCCTAAAGGACCCTAACCC).

We employed molecular dynamics simulations (MDS) to obtain a structural model of TiM9, as shown in Figure 1C. The MDS calculation results indeed show that it is not a typical duplex, stabilized by non-canonical hydrogen bonds (Figure S5). The two T bases at the 5' end are highly flexible and hard to form hydrogen bonds. The second base pair G•G is bonding via N-H ··N and N-H ··O (Figure S5A). Another G•G is paired by two hydrogen bonds of N-H ··O (Figure S5B). The A•A is paired via only one N-H ··N hydrogen bond (Figure S5C). There is a very short distance (~ 3.03 Å) between the two G•G base pairs, while that is longer (~ 3.68 Å) between the G•G and A•A base pairs. The average distance between bases is 3.41 Å, which is close to that of classic B-form DNA duplex. It suggests that the stacking interactions mainly between two G•G base pairs are another important contribution to the thermal stability of this non-canonical DNA duplex.

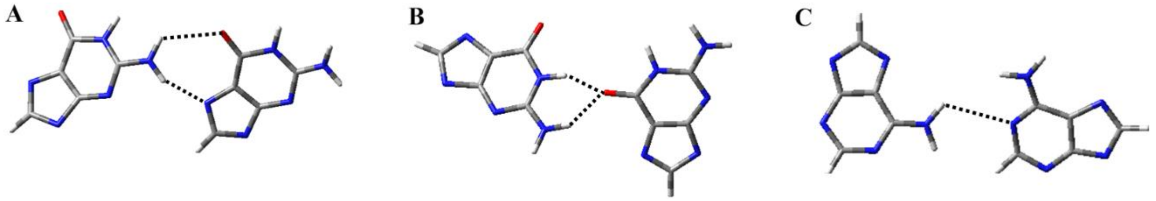


**Figure S6.** (A) G:G self-pairing via O···H−N and N···H−N to form hydrogen bonds. (B) G:G self-pairing via two hydrogen bonds formed by O···H−N. (C) A:A self-pairing via N···H−N to form a hydrogen bond. In all structures, N, O, C, H atoms are in blue, red, grey, white, respectively.

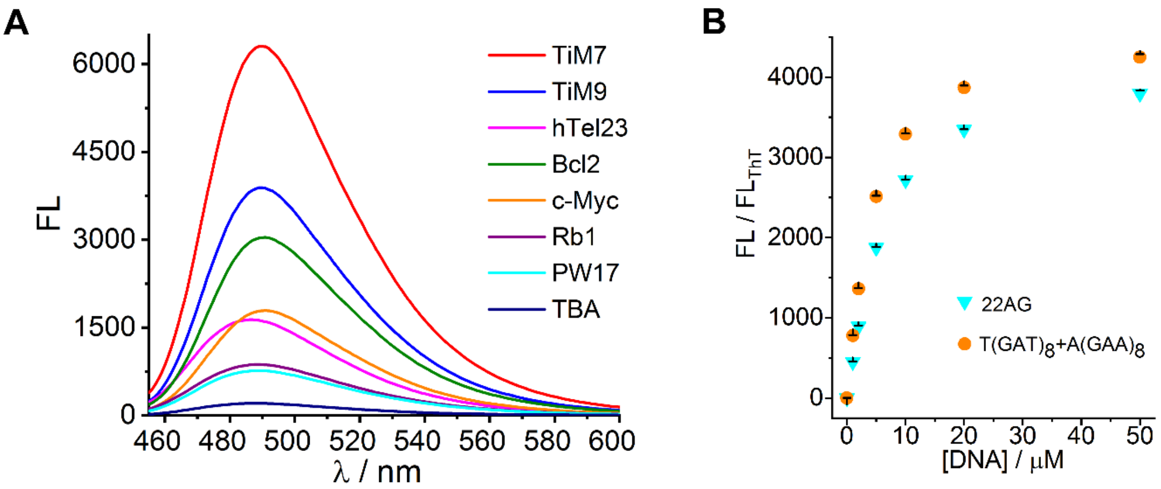


**Figure S7.** Parallel comparison between the ThT binding efficiencies of different DNA forms in pH 5 TAE buffer with 2 mM $MgCl_2$ and 100 mM KCl. (A) Comparison of 1 μM i-motif hybrids TiM7 and TiM9 with human native G4s or commonly used ones in the presence of 3 μM ThT. hTel23: TAGGGTTAGGGTTAGGGTTAGGG; Bcl2: GGGCGCGGGAGGAAGGGGGCGGG; c-Myc: AGGGTGGGGAGGGTGGGG; Rb1: CGGGGGGTTTTGGGCGGC; PW17: GGGTAGGGCGGGTTGGG; TBA: GGTTGGTGTGGTTGG. (B) Fluorescence titration of 3 μM ThT with different concentrations of 22AG (AGGGTTAGGGTTAGGGTTAGGG) (3) and a parallel duplex formed by $T(GAT)_8$ and $A(GAA)_8$ (13).

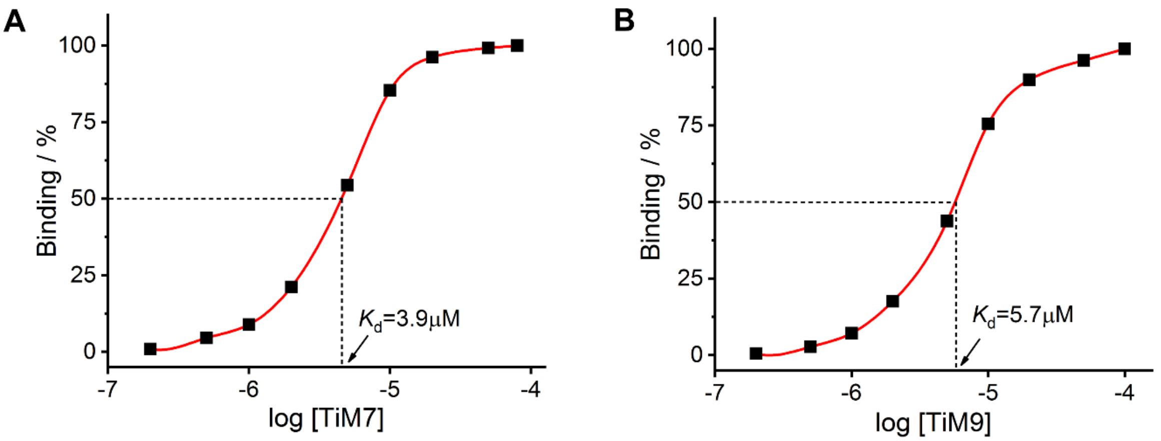


**Figure S8.** Affinity determination of TiM7 (A) and TiM9 (B) via fluorescence titration of 3 μM ThT with different DNA concentrations.

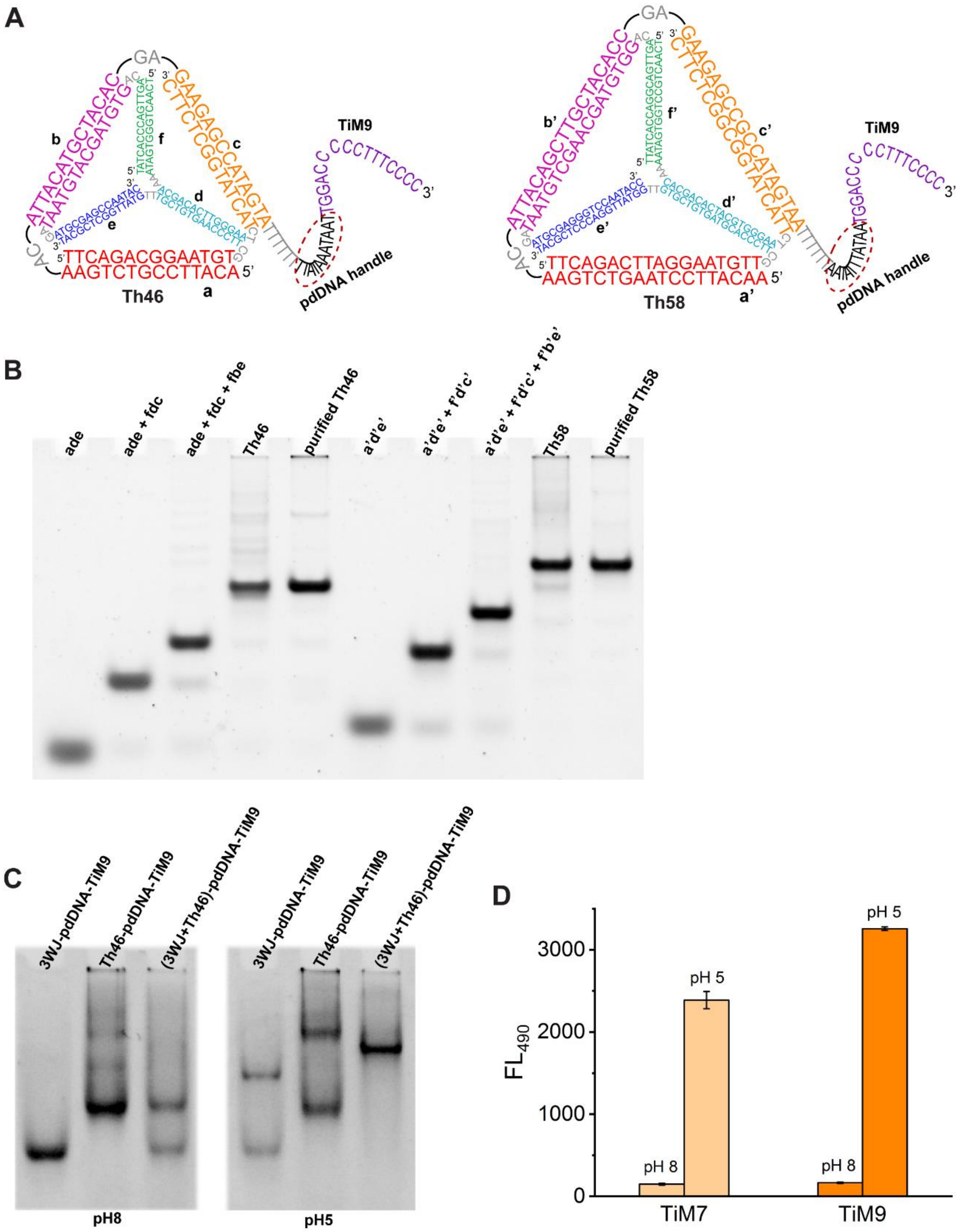


**Figure S9.** (A) Structural details of two DNA tetrahedra Th46 and Th58 tethered with TiM9 via a hetero pdDNA handle. (B) Step-by-step assembly of Th46 and Th58 in pH 8 TAE buffer with 2 mM $MgCl_2$ verified by native PAGE. (C) Gel electrophoretogram for modulating i-motif heterodimerization with a pdDNA handle that directs a three-way junction (3WJ) (12) and Th46 to form a hetero nanostructure in pH 5 TAE buffer with 2 mM $MgCl_2$. Here 3WJ is adopted in

place of Th58 due to a bigger difference between the electrophoretic mobility of their heterodimer and homodimers of 3WJ and Th46, which is easier for PAGE characterization. At pH 5, the bands of monomeric 3WJ and Th46 disappear entirely, and meanwhile a new band emerges who has a moderate mobility between the homodimers of 3WJ and Th46. It demonstrates that TiM9 can guide the assembly of Th46 and Th58 into a heterodimer rather than a homodimer as reported (12,15), illustrating its promising use as a modulator for functional biomolecular dimerization that may be applied to some physiological processes (16-20). (D) Comparison between TiM7 and TiM9 as the light-up systems for the pH-switched assembly of Th46 and Th58 shown in Figure 1B in the text. Although TiM7 shows a slightly higher enhancement on the ThT fluorescence than TiM9, the latter has a higher structural stability enough to link Th46 with Th58 to fully form a robust dimer at pH 5. So the TiM9 functionalized FNA system displays a better fluorescence performance for pH switching, ultimately, it's chosen in this study.

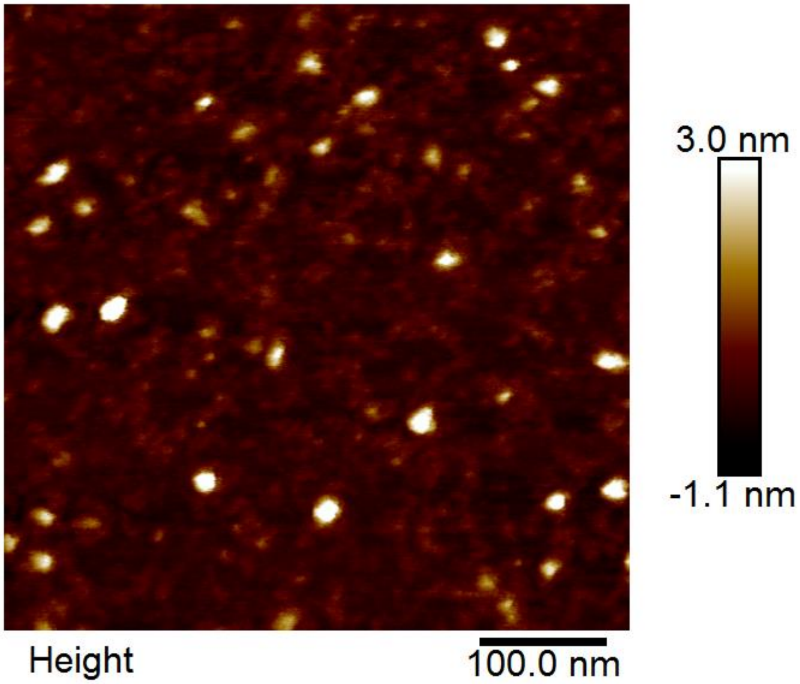


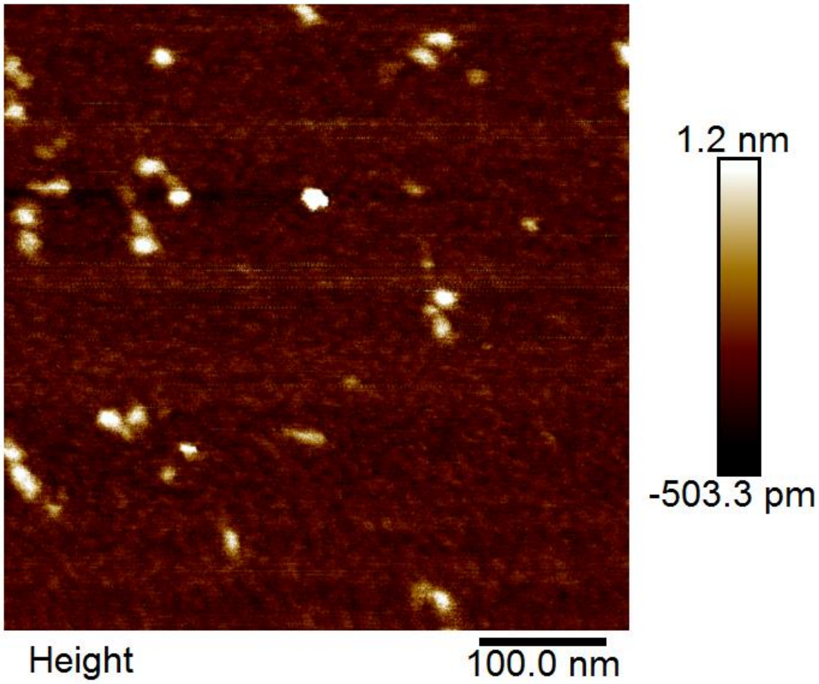


**Figure S10.** Entire high-resolution AFM image of monomeric (top) and dimeric (bottom) tetrahedrons shown in Figure 4.

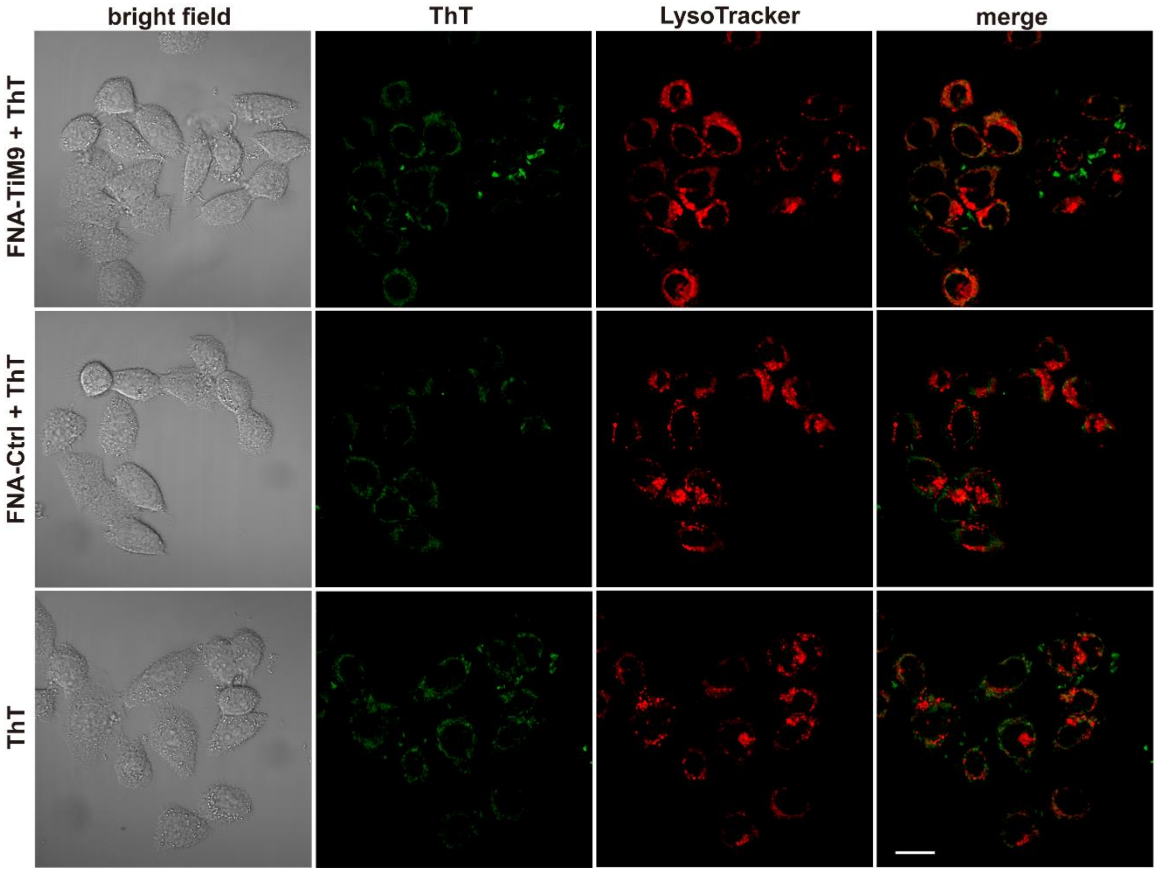


**Figure S11.** Confocal fluorescence images of the FNA/ThT system on cell membrane when pH is adjusted to pH 8. In the alkaline conditions, more ThT is found to enter cells and thus has bright fluorescence inside cells. The scale bar is 20 μm.

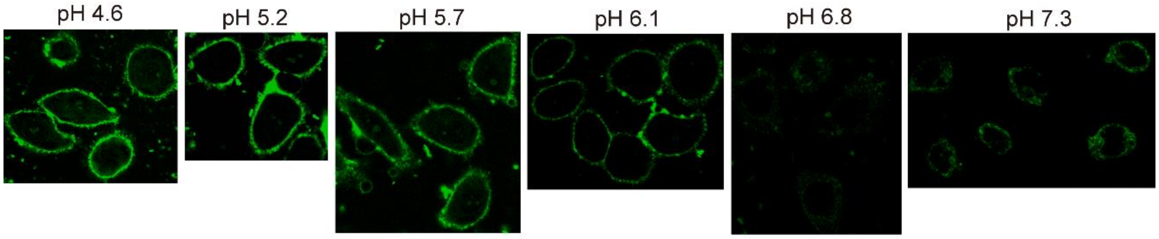


**Figure S12.** A large field of view of cell images for quantitative analysis of fluorescence intensity in Figure 5D.

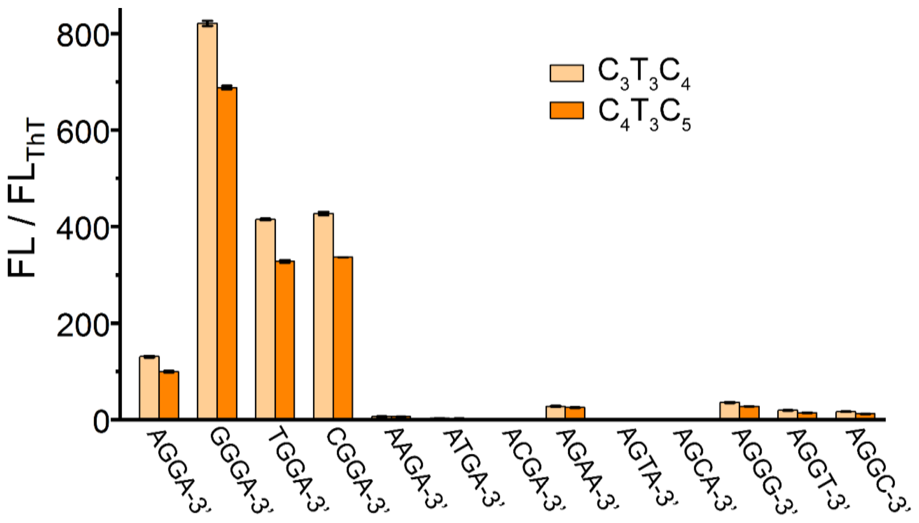


**Figure S13.** Addressing the ThT-interactive preference site via one-by-one base substitution for the 3'-AGGA overhang of $C_3T_3C_4$ and $C_4T_3C_5$.

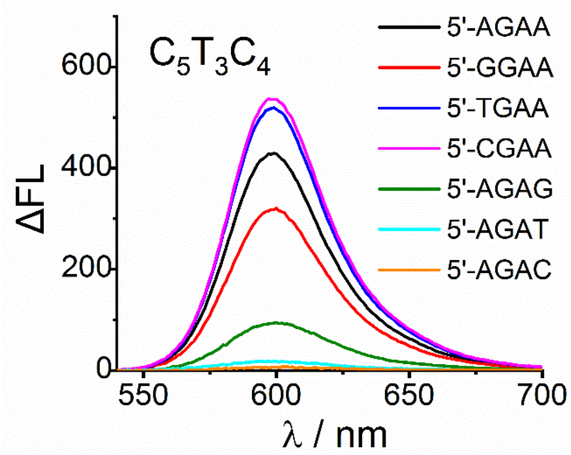


**Figure S14.** Comparison between the binding efficiency of different 5'-overhang appended to $C_5T_3C_4$ (1 μM) in the presence of 3 μM DSMB in pH 5 TAE buffer with 2 mM $MgCl_2$.

**Table S1.** Sequences of tetrahedrons used in this study.

| Name | Sequences |
|---|---|
| Th46-ade | TTCAGACGGAATGTGCTTCCCAAGTGTCGTTTGTATTGGCTCGCAT |
| Th46-fdc | TCAACTGGGTGATAAAACGACACTTGGGAATCTACTATGGCTCTTC |
| Th46-fbe | TATCACCCAGTTGACAGTGTAGCATGTAATAGATGCGAGCCAATAC |
| Th46-abc-ps-TiM9 | ACATTCCGTCTGAAACATTACATGCTACACGAGAAGAGCCATAGTATTTTTTTTATAAATAATTGGAC CCCCTTTCCCC |
| Th46-abc-ps-Ctrl | ACATTCCGTCTGAAACATTACATGCTACACGAGAAGAGCCATAGTATTTTTTTTATAAATAATTTTAT TTTCTTTCTTT |
| Th58-a’d’e’ | TTCAGACTTAGGAATGTTGCTTCCCACGTAGTGTCGTGTTGGTATTGGACCCTCGCAT |
| Th58-f’d’c’ | TCAACTGCCTGGTGATAAAACACGACACTACGTGGGAATCTTACTATGGCGGCTCTTC |
| Th58-f’b’e’ | TTATCACCAGGCAGTTGACAGGTGTAGCAAGCTGTAATAGATGCGAGGGTCCAATACC |
| Th58-a’b’c’-ps-TiM9 | AACATTCCTAAGTCTGAAACATTACAGCTTGCTACACCGAGAAGAGCCGCCATAGTAATTTTTTAAT ATTTATAATGGACCCCCTTTCCCC |
| Th58-a’b’c’-ps-Ctrl | AACATTCCTAAGTCTGAAACATTACAGCTTGCTACACCGAGAAGAGCCGCCATAGTAATTTTTTAAT ATTTATAATTTATTTTCTTTCTTT |
| Th46-ade-chol | TTCAGACGGAATGTGCTTCCCAAGTGTCGTTTGTATTGGCTCGCAT-cholesteryl |
| Th46-fdc-chol | TCAACTGGGTGATAAAACGACACTTGGGAATCTACTATGGCTCTTC-cholesteryl |
| Th46-fbe-chol | TATCACCCAGTTGACAGTGTAGCATGTAATAGATGCGAGCCAATAC-cholesteryl |
| Th58-a’d’e’-chol | TTCAGACTTAGGAATGTTGCTTCCCACGTAGTGTCGTGTTGGTATTGGACCCTCGCAT-cholesteryl |
| Th58-f’d’c’-chol | TCAACTGCCTGGTGATAAAACACGACACTACGTGGGAATCTTACTATGGCGGCTCTTC-cholesteryl |
| Th58-f’b’e’-chol | TTATCACCAGGCAGTTGACAGGTGTAGCAAGCTGTAATAGATGCGAGGGTCCAATACC-cholesteryl |

## References


1. Arthanari, H., Basu, S., Kawano, T.L. and Bolton, P.H. (1998) Fluorescent dyes specific for quadruplex DNA. *Nucleic Acids Res.*, **26**, 3724-3728.
2. Nicoludis, J.M., Miller, S.T., Jeffrey, P.D., Barrett, S.P., Rablen, P.R., Lawton, T.J. and Yatsunyk, L.A. (2012) Optimized end-stacking provides specificity of N-methyl mesoporphyrin IX for human telomeric G-quadruplex DNA. *J. Am. Chem. Soc.*, **134**, 20446-20456.
3. Mohanty, J., Barooah, N., Dhamodharan, V., Harikrishna, S., Pradeepkumar, P.I. and Bhasikuttan, A.C. (2013) Thioflavin T as an Efficient Inducer and Selective Fluorescent Sensor for the Human Telomeric G-Quadruplex DNA. *J. Am. Chem. Soc.*, **135**, 367-376.
4. Allain, C., Monchaud, D. and Teulade-Fichou, M.P. (2006) FRET templated by G-quadruplex DNA: a specific ternary interaction using an original pair of donor/acceptor partners. *J. Am. Chem. Soc.*, **128**, 11890-11893.
5. Kang, B.H., Gao, Z.F., Li, N., Shi, Y., Li, N.B. and Luo, H.Q. (2016) Thiazole orange as a fluorescent probe: Label-free and selective detection of silver ions based on the structural change of i-motif DNA at neutral pH. *Talanta*, **156**, 141-146.
6. Lee, I.J., Patil, S.P., Fhayli, K., Alsaiari, S. and Khashab, N.M. (2015) Probing structural changes of self assembled i-motif DNA. *Chem. Commun.*, **51**, 3747-3749.
7. Liu, L., Shao, Y., Peng, J., Huang, C., Liu, H. and Zhang, L. (2014) Molecular rotor-based fluorescent probe for selective recognition of hybrid G-quadruplex and as a K+ sensor. *Anal. Chem.*, **86**, 1622-1631.

8. Feng, G., Luo, C., Yi, H., Yuan, L., Lin, B., Luo, X., Hu, X., Wang, H., Lei, C., Nie, Z. *et al.* (2017) DNA mimics of red fluorescent proteins (RFP) based on G-quadruplex-confined synthetic RFP chromophores. *Nucleic Acids Res.*, **45**, 10380-10392.
9. Kreig, A., Calvert, J., Sanoica, J., Cullum, E., Tipanna, R. and Myong, S. (2015) G-quadruplex formation in double strand DNA probed by NMM and CV fluorescence. *Nucleic Acids Res.*, **43**, 7961-7970.
10. Ai, J., Li, T., Li, B., Xu, Y., Li, D., Liu, Z. and Wang, E. (2012) In situ labeling and imaging of cellular protein via a bi-functional anticancer aptamer and its fluorescent ligand. *Anal. Chim. Acta*, **741**, 93-99.
11. Nonin, S., Phan, A.T. and Leroy, J.L. (1997) Solution structure and base pair opening kinetics of the i-motif dimer of d(5mCCTTTACC): a noncanonical structure with possible roles in chromosome stability. *Structure*, **5**, 1231-1246.
12. Shi, L., Peng, P., Du, Y. and Li, T. (2017) Programmable i-motif DNA folding topology for a pH-switched reversible molecular sensing device. *Nucleic Acids Res.*, **45**, 4306-4314.
13. Liu, S., Peng, P., Wang, H., Shi, L. and Li, T. (2017) Thioflavin T binds dimeric parallel-stranded GA-containing non-G-quadruplex DNAs: a general approach to lighting up double-stranded scaffolds. *Nucleic Acids Res.*, **45**, 12080–12089.
14. Xu, L.J., Hong, S.N., Sun, N., Wang, K.W., Zhou, L., Ji, L.Y. and Pei, R.J. (2016) Berberine as a novel light-up i-motif fluorescence ligand and its application in designing molecular logic systems. *Chem. Commun.*, **52**, 179-182.
15. Peng, P., Du, Y., Zheng, J., Wang, H. and Li, T. (2019) Reconfigurable Bioinspired Framework Nucleic Acid Nanoplatform Dynamically Manipulated in Living Cells for Subcellular Imaging. *Angew. Chem. Int. Ed.*, **58**, 1648-1653.
16. Talanian, R.V., McKnight, C.J. and Kim, P.S. (1990) Sequence-specific DNA binding by a short peptide dimer. *Science*, **249**, 769-771.
17. Kalodimos, C.G., Biris, N., Bonvin, A.M., Levandoski, M.M., Guennuegues, M., Boelens, R. and Kaptein, R. (2004) Structure and flexibility adaptation in nonspecific and specific protein-DNA complexes. *Science*, **305**, 386-389.
18. Sauerwald, A., Sandin, S., Cristofari, G., Scheres, S.H., Lingner, J. and Rhodes, D. (2013) Structure of active dimeric human telomerase. *Nat. Struct. Mol. Biol.*, **20**, 454-460.
19. Bandukwala, H.S., Wu, Y., Feuerer, M., Chen, Y., Barboza, B., Ghosh, S., Stroud, J.C., Benoist, C., Mathis, D., Rao, A. *et al.* (2011) Structure of a domain-swapped FOXP3 dimer on DNA and its function in regulatory T cells. *Immunity*, **34**, 479-491.
20. Morii, T., Saimei, Y., Okagami, M., Makino, K. and Sugiura, Y. (1997) Factors governing the sequence-selective DNA binding of geometrically constrained peptide dimers. *J. Am. Chem. Soc.*, **119**, 3649-3655.
